# Rictor regulates the vasculogenic mimicry of melanoma *via* the AKT‐MMP‐2/9 pathway

**DOI:** 10.1111/jcmm.13268

**Published:** 2017-07-12

**Authors:** Xingmei Liang, Ran Sun, Xiulan Zhao, Yanhui Zhang, Qiang Gu, Xueyi Dong, Danfang Zhang, Junying Sun, Baocun Sun

**Affiliations:** ^1^ Department of Pathology Tianjin Medical University Tianjin China; ^2^ Department of Surgery Tianjin Hospital of ITCWM Nankai Hospital Tianjin China; ^3^ Department of Pathology General Hospital of Tianjin Medical University Tianjin China; ^4^ Department of Pathology Cancer Hospital of Tianjin Medical University Tianjin China

**Keywords:** Rictor, AKT, MMP‐2, MMP‐9, vasculogenic mimicry

## Abstract

Vasculogenic mimicry (VM)‐positive melanomas are usually associated with poor prognosis. Rictor, the key component of the rapamycin‐insensitive complex of mTOR (mTORC2), is up‐regulated in several cancers, especially in melanomas with poor prognosis. The aim of this study was to investigate the role of Rictor in the regulation of VM and the mechanism underlying this possible regulation. VM channels were found in 35 of 81 tested melanoma samples and high Rictor expression correlated with VM structures. Moreover, Kaplan–Meier survival curves indicated that VM structures and high Rictor expression correlated with shorter survival in patients with melanoma. *In vitro*, Rictor knockdown by short hairpin RNA (shRNA) significantly inhibited the ability of A375 and MUM‐2B melanoma cells to form VM structures, as evidenced by most tubes remaining open. Cell cycle analysis revealed that Rictor knockdown blocked cell growth and resulted in the accumulation of cells in G2/M phase, and cell migration and invasion were greatly affected after Rictor down‐regulation. Western blotting assays indicated that down‐regulating Rictor significantly inhibited the phosphorylation of AKT at Ser^473^ and Thr^308^, which subsequently inhibited the expression and activity of downstream MMP‐2/9, as confirmed by real‐time PCR and gelatin Zymography. MK‐2206, a small‐molecule inhibitor of AKT, similarly inhibited the activity of AKT and secretion of MMP‐2/9, further supporting that Rictor down‐regulation inhibits the phosphorylation of AKT and activity of downstream MMP‐2/9 to affect VM formation. In conclusion, Rictor plays an important role in melanoma VM *via* the Rictor—AKT—MMP‐2/9 signalling pathway.

## Introduction

Melanoma is the most serious type of skin cancer and is associated with an aggressive clinical course and poor prognosis. Previous studies have confirmed VM in several malignant tumours with poor survival 1, such as ovarian cancer 2, breast cancer 3, lung cancer 4, prostate cancer 5, glioma 6 and renal cell carcinoma 7. VM describes the ability of highly invasive melanoma cells to express endothelium‐associated genes and form extracellular matrix (ECM)‐rich vasculogenic‐like channels in three‐dimensional culture. VM, independent of endothelium‐dependent vessels, invariably indicates poor prognosis and survival in the clinic 8. In addition, traditional anti‐angiogenesis therapy cannot inhibit VM lumen formation by aggressive cutaneous and uveal melanoma cells 9. In recent years, many researchers have focused on the molecular mechanism underlying this unique process, and several critical modulating molecules have been confirmed to be involved in the formation of the VM network, such as VE‐cadherin, PI3K, AKT, HIF, Twist1, MT1‐MMP, MMP‐2 and MMP‐9 10, 11, 12. Moreover, the rapamycin‐insensitive companion of mTOR (Rictor), first identified in 2004 13, is overexpressed in many types of tumors and is usually associated with poor prognosis in the clinic 14, 15. A recent study found that up‐regulation of Rictor contributed to the invasion and metastasis of cancer cells 16, which are requirements for VM. However, the role of Rictor in cancer cell VM formation is largely unknown.

The mammalian target of rapamycin (mTOR) plays an important role in cell proliferation and survival 17, 18. Based on the sensitivity to rapamycin treatment, mTOR is separated into two distinct multiprotein complexes, a rapamycin‐sensitive mTOR complex 1 (mTORC1) and a Rictor‐containing rapamycin‐insensitive mTOR complex 2 (mTORC2). The two complexes are homologous and highly conserved in sequence 19. A recent study verified that mTORC1 is involved in the VM of glioma *via* hypoxia‐inducible factor‐1 alpha 6, indicating that mTORC2 may also play a role in VM. Moreover, the mTORC2 complex can phosphorylate and activate AKT at Ser^473^ 20, 21, which is the central component in the PI3K‐AKT pathway 22, and regulate the expression of downstream matrix metalloproteinases (MMPs) 23, 24, 25. MMPs have also been confirmed to be involved in angiogenesis 26 and VM 10. Therefore, mTORC2 is proposed to regulate VM *via* AKT and MMPs. Rictor is a key component of mTORC2 and is required for mTORC2 function 13, and Rictor knockdown significantly inhibits the activation of AKT 20 and affects the migration, invasion and proliferation of cancer cells, which are essential conditions for cells to form VM structures 16. In addition, Rictor is involved in PI3K/AKT pathway regulation in melanocytes and melanoma 27, while PI3K regulates membrane MMP‐1 and MMP‐2 activity during melanoma cell VM 28. Considering the function of Rictor in mTORC2 and the PI3K/AKT pathway, we hypothesize that Rictor may regulate the phosphorylation and activation of AKT to affect VM by regulating the expression and activation of MMP‐2/9 in melanoma. In the current report, we investigate the correlation between Rictor and VM in human melanoma tissues and examine the role of Rictor in cell motility and VM in A375 and MUM‐2B melanoma cell lines.

## Materials and methods

### Cells and cell culture

The human cutaneous (A375) and human uveal (MUM‐2B) melanoma cancer cells were obtained from China Infrastructure of Cell Line Resources (Beijing, China). A375 melanoma cells were cultured in DMEM (Hyclone), and MUM‐2B melanoma cells were grown in RPMI 1640 (Hyclone), both supplemented with 10% foetal bovine serum (FBS; Gibco, New York, USA), and penicillin/streptomycin (100 U/ml/100 μg/ml) at 37°C in 5% CO2.

### Main reagents and antibodies

The following primary antibodies were used: antibodies against Rictor (ab70374), MMP‐2 (ab37150) and MMP‐9 (ab76003) from Abcam (Cambridge, USA); antibodies against phospho‐AKT (S473) (#9721) and phospho‐AKT (T308) (#13038) from Cell Signaling Technology; and antibodies against AKT (AF6259), phospho‐CDK2 (Thr160) (AF3237), phospho‐Histone H3.1 (Ser10) (AF3358) and β‐actin (T0022) from Affinity Biosciences. HRP‐conjugated goat anti‐rabbit IgG and anti‐mouse IgG secondary antibodies were obtained from Santa Cruz (Dallas, TX, USA). Gelatin (G7041) was purchased from Sigma‐Aldrich (St. Louis, MO, USA). MK‐2206, 8‐[4‐(1‐aminocyclobutyl)phenyl]‐9‐phenyl‐1,2,4‐triazolo [3,4‐f] 1, 6naphthyridin‐3(2H)‐one hydrochloride [1:1], was obtained from Selleck (Shanghai, China). 3‐(4,5‐dimethyl‐2‐thiazolyl)‐2,5‐diphenyl‐2‐H‐tetrazolium bromide (MTT) was purchased from Sigma‐Aldrich (St. Louis, MO).

### Immunohistochemical staining and assessment

Eighty‐one paraffin‐embedded melanoma tissue specimens and their clinical pathological data were obtained from the Cancer Institute and Hospital of Tianjin Medical University between 1999 and 2010. Each specimen was reviewed by a pathologist, and the use of patient specimens was approved by the Institutional Research Committee. The experimental procedures and scoring of the immunohistochemical assay were performed as described in our previous report 29. The following antibodies and dilutions were employed: Rictor (1:400), AKT (1:200), MMP‐2 (1:200) and CD34 (1:50). PBS was used in place of the primary antibodies for all negative controls. Periodic acid‐Schiff (PAS) staining was performed after CD34 immunohistochemical staining, and normal gastric mucosa was selected as the positive control. PAS‐positive channels exclusively lined by tumour cells without CD34‐stained endothelial cells indicated VM, where red blood cells were present.

### shRNA and plasmid transfection

To further assess the role of Rictor in melanoma cells, we used a shRNA‐based technique to specifically silence Rictor expression in A375 and MUM‐2B cells. Rictor down‐regulation was mediated by lentiviral infection using OmicsLink shRNA expression clones (catalogue no. HSH006478‐LVRU6GP; GeneCopoeia, Rockville, MD, USA). A negative control (catalogue no. CSHCTR001‐LVRU6GP) was also transfected. Specifically, 4 shRNA target sequences against Rictor (#1: GGTTAGTAGTAGAAAGTTCAA; #2: GCTACTTAGAAGATCTAGTAA; #3: GGGTCTAGTTGAAGTGATAAC; and #4: CCCGAGAACCTTCTGATAACT) and a scrambled sequence were synthesized by GeneCopoeia. Transfection was performed with the Lenti‐Pac HIV packaging kit (catalogue no. HPK‐LvTR‐20; GeneCopoeia) in accordance with the manufacturer's instructions. At 48 hrs after transfection, a fluorescence microscope (Nikon, Tokyo, Japan) was used to examine the transfection efficiency. Subsequently, we harvested the transfected cells for further experiments.

### Three‐dimensional (3D) cultures

For this assay, 96‐well plates were coated with 35 μl of Matrigel matrix, pre‐treated on ice for 20 min. and incubated for 1 hr at 37°C. A suspension of A375 or MUM‐2B cells containing 2 × 10^4^ cells was seeded onto the gel and incubated at 37°C for 12 hrs. Subsequently, each well was observed and filmed under a phase‐contrast microscope (100×).

### Cell proliferation assay

To better understand the effect of Rictor on cell proliferation, MTT assays were conducted. Rictor‐silenced and control cells were plated in 96‐well plates at 800 cells/well and incubated at 37°C in 5% CO_2_. Subsequently, 10 μl of MTT reagent (10 mg/ml, Sigma‐Aldrich, St Louis, MO, USA) was added to each well, and the cells were incubated for 4 hrs. The medium was then discarded, and 100 μl of dimethylsulphoxide (DMSO) was added to each well. The plate was then gently shaken until the purple crystals dissolved. Subsequently, the absorbance of each well was measured at 490 nm with a Spectra Max M2 spectrophotometer (Molecular Devices, Sunnyvale, CA, USA).

### Cell cycle analysis

A375 and MUM‐2B cells pre‐treated with scr or shRictor were plated in 6‐well plates to form a monolayer at approximately 80% confluence. The cells were then collected, fixed with 70% ethanol overnight at 4°C and stained with PI protected from light using a Cycle TEST PLUS DNA Reagent Kit (BD Pharmingen™, BD Biosciences, San Jose, CA, USA). Subsequently, the samples were subjected to flow cytometry with a BD FACSVerse flow cytometer (BD Biosciences, San Jose, CA, USA), and 20,000 events were collected from each sample. Data acquisition was performed using Cell‐Quest software, and the cell cycle distribution was calculated using BD FACSuite software (BD Biosciences). The number of gated cells in G1, G2/M or S phase was expressed as a percentage.

### Cell migration and invasion assay

Cell migration and invasion were evaluated by transwell assays. First, 100 μl of serum‐free cell suspension containing 1 × 10^5^ cells was plated into the upper chamber, which had been coated with Matrigel matrix (BD Biosciences, San Jose, CA, USA) for invasion assays or remained uncoated for migration assays. Subsequently, 500 μl of medium containing 10% FBS was added to the lower chamber. After 24 hrs (migration) and 48 hrs (invasion), the migrating cells were fixed with cold methanol and stained with 0.4% crystal violet solution, and the number of migrating cells was counted under an inverted light microscope (Nikon) at 100× magnification. Each experiment was performed three times.

### Wound‐healing assay

Wound‐healing assays (conventional scrape motility assays) were performed according to the protocol described by Zhang *et al*. 30. A375 and MUM‐2B cells pre‐treated with scrambled control oligomer (scr) or shRictor were cultured in 6‐well culture plates in a monolayer at 80–90% confluence. Then, an even trace was lined out in the middle of each well using a 10‐μl pipette tip. The cells were then incubated in serum‐free medium at 37°C in 5% CO_2_, and the wounds were photographed and measured. The rate of wound closure was monitored at intervals by measuring the ratio of the migration distance to that at 0 hr until the wounds were occluded.

### Adhesion assay

Adhesion assays were performed as described previously 31. Briefly, A375 and MUM‐2B melanoma cells were pre‐treated with scr or shRictor and washed three times with binding medium (BM) (DMEM, 0.1% BSA and 25 mM HEPES), suspended at a density of 2 × 10^5^ cells/ml in serum‐free medium and then incubated at 37°C in a 5% CO_2_ incubator for 20 min. Subsequently, 1 ml of the cell suspension was promptly placed in a 35‐mm dish containing a dried glass coverslip coated with 10 ng/ml fibronectin and incubated at 4°C overnight. The cells were gently washed, fixed and counted at 100× magnification in five separate fields under a light microscope.

### Western blotting analysis

The cells were lysed on ice for 30 min., and 15 μg of protein per sample was separated using 10% SDS‐PAGE. The separated proteins were then transferred onto PVDF membranes (Millipore, Darm stadt, Germany). For phospho‐Akt (Ser^473^) antibody, the membrane was incubated with diluted antibody in 5% w/v BSA, 1 × TBS and 0.1% Tween‐20 at 4°C overnight. For staining with other antibodies, the membranes were blocked with 5% non‐fat milk for 1 hr at room temperature and incubated with diluted primary antibodies overnight at 4°C. The antibodies and dilution factors were as follows: Rictor (1:4000), AKT (1:1000), p‐AKT Ser^473^ (1:1000), p‐AKT Thr^308^ (1:1000), MMP‐2 (1:1000) and MMP‐9 (1:500). Secondary antibodies (1:2000) were incubated for 2 hrs at room temperature the following day. A C‐Digit Blotting Scanner (Gene Company, Beijing, China) was used to photograph and analyse bands using ImageJ software.

### Real‐time PCR

Total RNA extracted from A375 and MUM‐2B cells was isolated with TRNzol‐A^+^ Reagent (TIANGEN, Beijing, China), and cDNA was synthesized using the PrimeScript™ RT reagent Kit with gDNA Eraser (TaKaRa, Dalian,China). The following real‐time PCR primers were used: Rictor (forward: 5′‐TTTCGGGGATTTCTGGATG‐3′, reverse: 5′‐AAAGCCCAGTCTCATGACATT‐3′), MMP‐2 (forward: 5′‐AAGGATGGCAAGTACGGCTT‐3′, reverse: 5′‐CGCTGGTACAGCTCTCATACTT‐3′), MMP‐9 (forward: 5′‐ACCTCGAACTTTGACAGCGAC‐3′, reverse: 5′‐GAGGAATGATCTAAGCCCAGC‐3′) and β‐actin (forward: 5′‐GGCCGGGACCTGACTGACTAC‐3′, reverse: 5′‐GCCGCCAGACAGCACTGTGTT‐3′). PCR was performed using real‐time PCR Master Mix (SYBR Green) according to the manufacturer's instructions. Signals were detected with an ABI 7500 Real‐Time PCR System (Applied Biosystems, California, USA).

### Zymography assays

To assess MMP‐2 and MMP‐9 activity in A375 and MUM‐2B cells, we collected serum‐free conditioned medium for SDS‐PAGE using a 10% polyacrylamide gel containing 0.01% w/v gelatin. The gels were loaded with 30 μl of medium from each sample, and electrophoresis was conducted in ice water at 120 V for 3 hrs. After electrophoresis, the gels were equilibrated 4 times in 50 mM Tris–HCl (pH 7.5) containing 2.5% Triton X‐100 for 30 min. each with gentle shaking to remove SDS. Subsequently, the gels were incubated in substrate buffer (1 M Tris‐HCl PH 7.5, 0.1 M CaCl_2_ and 100 mM ZnCl2) for 42 hrs at 37°C and then stained with Coomassie Brilliant Blue R250 for 2 hrs with gentle shaking. The gels were subsequently washed until clear bands appeared.

### AKT inhibitor, MK‐2206

MK‐2206, a small‐molecule and allosteric inhibitor of AKT, was dissolved in DMSO and used to treat cells for 24 hrs at concentrations of 2, 4 and 8 μM 32, 33. The cells were then collected and lysed for Western blotting assays to measure the phosphorylation of AKT and expression of MMP‐2/9, while supernate media was collected for zymography assay to evaluate the activity of MMP‐2/9.

### Statistical analysis

All data were analysed with SPSS version 17.0 (SPSS, Chicago, IL, USA). The measured data are expressed as the mean ± standard deviation (S.D.) or standard error of the mean (S.E.M.) A *P* value of <0.05 was defined as significant. Differences between two groups were assessed using Student's t‐test, whereas multiple groups were compared by anova. Kaplan–Meier survival curve analysis was also performed. Differences in survival curves were evaluated using the log‐rank test.

## Results

### Rictor is overexpressed in invasive melanoma and correlates with VM

**Figure 1:**
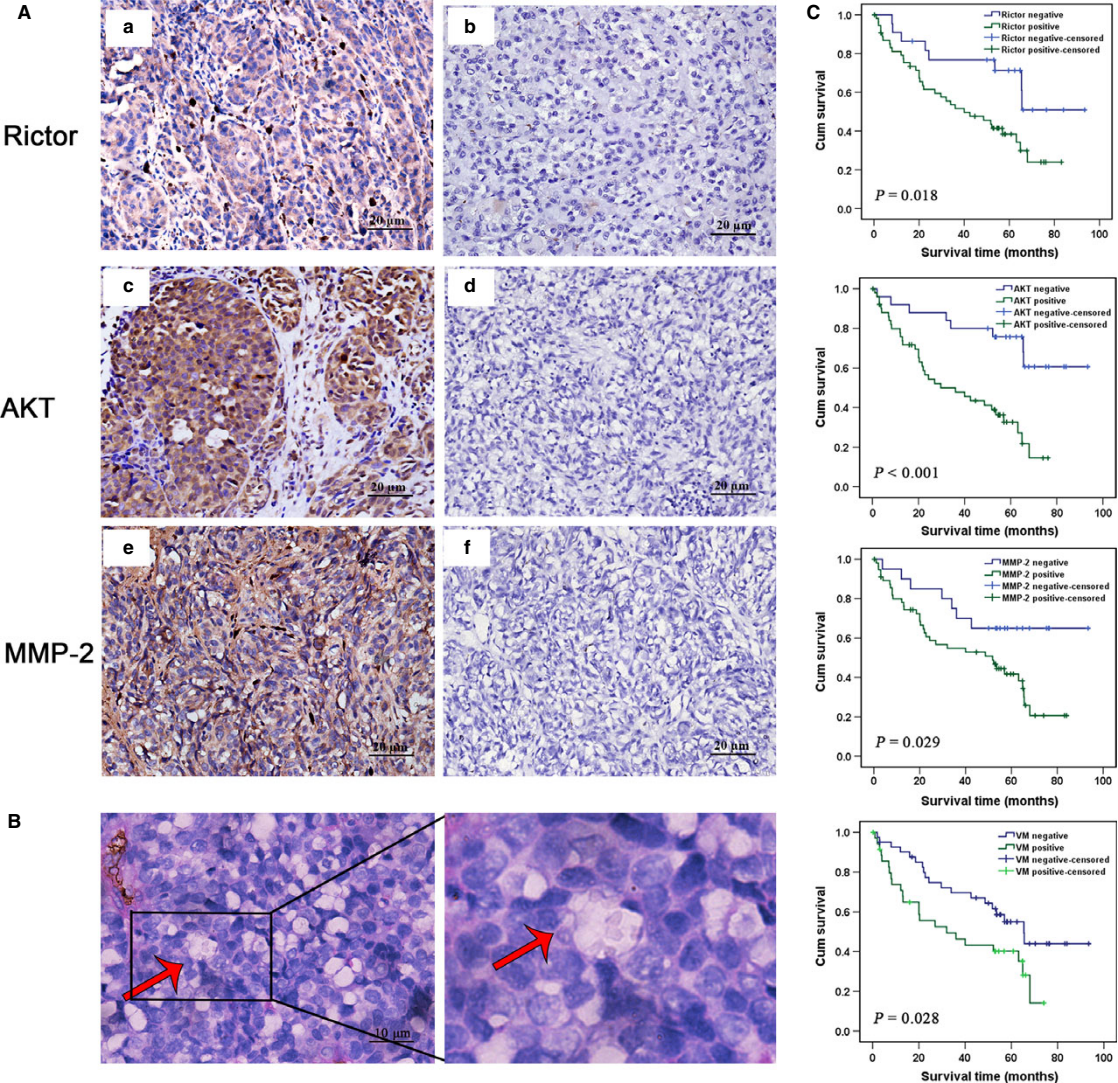
Rictor is overexpressed in invasive melanoma and correlated with VM. (**A**), IHC staining of (a) Rictor, (c) AKT, (e) MMP‐2 expression. b, d, f were negative controls without primary antibodies for Rictor, AKT, MMP‐2, respectively. (**B**) Phenomena of VM (red arrow, CD34‐negative and PAS‐positive) in melanoma. (**C**) Kaplan–Meier survival curve demonstrating that high Rictor, AKT, MMP‐2 expression and VM positive are significantly related to poor prognosis (all *P* < 0.05).

The expression of Rictor was detected by immunohistochemistry in 81 melanoma tissue samples. Specifically, 57 samples were defined as positive staining, among which 36 cases were accompanied with recurrence or metastasis, suggesting that the expression of Rictor is elevated during melanoma tumorigenesis (*P =* 0.014; Table 1, Fig. 1Aa). In addition, melanoma patients along with Rictor overexpression always have a poor prognosis and survival, resembling to those with VM formation (Fig. 1C). VM tubes with PAS‐positive and CD34‐negative staining were observed in 30 samples with Rictor up‐regulation but only five samples with Rictor‐negative staining (*P =* 0.002; Table 1, Fig. 1B), indicating that the expression of Rictor closely correlates with VM. Moreover, 47 and 46 cases of the 57 samples with elevated Rictor expression stained positive for AKT and MMP‐2, respectively (*P =* 0.000 and *P =* 0.036, respectively; Table 1, Fig. 1Ab and Ac), which may serve as downstream effectors and have been confirmed to be involved in VM formation 10, 26 (*P =* 0.007 and *P =* 0.002; Table 2). Taken together, the above results indicate that the expression of Rictor is up‐regulated in melanoma and may be associated with VM *via* AKT/MMPs.

**Table 1:** The correlation of Rictor with the clinicopathological parameter of melanoma

Variables	Cases	Rictor expression		χ^2^	*P*‐value
		Positive(57)	Negative(24)		
Gender					
Male	54	38	16	0.0000	1.000
Female	27	19	8		
Age(years)					
<55	22	15	7	0.069	0.792
≥55	59	42	17		
Tumour size (cm^3^)					
<7.8	52	37	15	0.043	0.836
≥7.8	29	20	9		
Pathological grade					
I–II	41	25	16	3.515	0.061
III–IV	40	32	8		
Recurrence or Metastasis					
Yes	44	36	8	6.054	0.014
No	37	21	16		
VM					
Positive	35	31	4	9.792	0.002
Negative	46	26	20		
AKT					
Positive	54	47	7	21.528	<0.001
Negative	27	10	17		
MMP‐2					
Positive	60	46	14	4.400	0.036
Negative	21	11	10		

**Table 2:** The correlation of VM with the clinicopathological parameter of melanoma

Variables	Cases	VM		χ^2^	*P*‐value
		Positive(35)	Negative(46)		
Gender					
Male	54	23	31	0.025	0.874
Female	27	12	15		
Age(years)					
<55	22	11	11	0.567	0.451
≥55	59	24	35		
Tumour size (cm^3^)					
<7.8	52	22	30	0.048	0.826
≥7.8	29	13	16		
Pathological grade					
I–II	41	15	26	1.485	0.223
III–IV	40	20	20		
Recurrence or Metastasis					
Yes	44	24	20	5.044	0.025
No	37	11	26		
Rictor					
Positive	57	31	26	9.792	0.002
Negative	24	4	20		
AKT					
Positive	54	29	25	7.270	0.007
Negative	27	6	21		
MMP‐2					
Positive	60	32	28	9.665	0.002
Negative	21	3	18		

### Down‐regulation of Rictor inhibits VM channels formation *in vitro*

**Figure 2:**
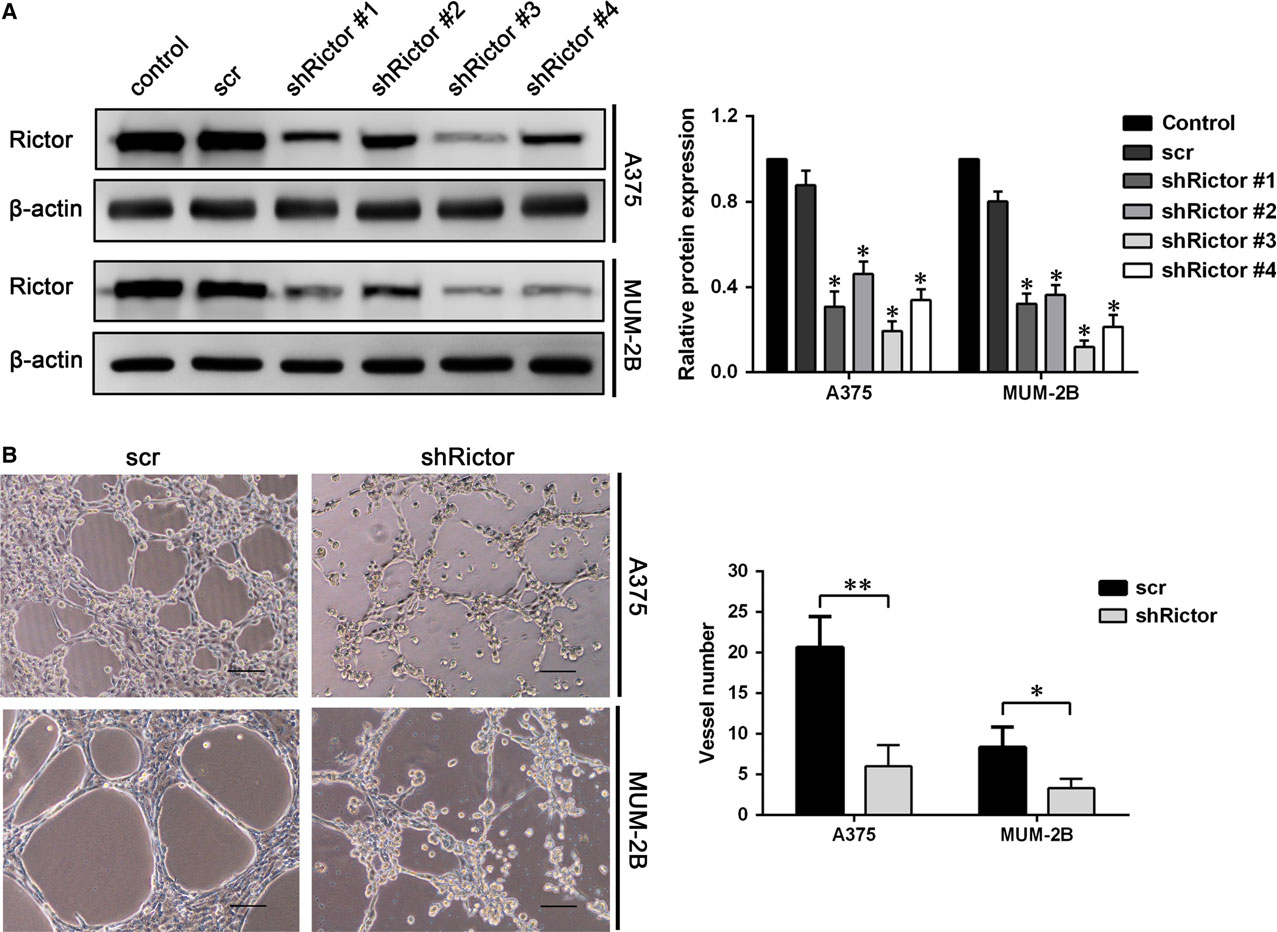
Down‐regulation of Rictor inhibited VM formation *in vitro*. (**A**) Rictor was knocked down by shRNA using four different sequences in A375 and MUM‐2B cells (*P < 0.05). (**B**) Rictor down‐regulation inhibited VM formation on Matrigel by A375 and MUM‐2B cells (bar 100 μm, *P < 0.05, **P < 0.01).

We hypothesize that Rictor plays an important role in the VM of melanoma cells. To test this hypothesis, four independent shRNAs (#1, #2, #3 and #4) were designed to target Rictor, and a scrambled sequence was used as a negative control. Transient transfection with each of the four shRNAs specifically down‐regulated Rictor expression in A375 and MUM‐2B cells, whereas transfection with the scrambled sequence did not (Fig. 2A). One of the shRNA sequences (#3) was selected to generate stable clones and served as a representative for later results. To exclude false‐positive results, the results of shRictor#1 were shown in supplementary material. The ability of melanoma cells to form capillary‐like tubes was evaluated *in vitro* by seeding the cells on Matrigel‐coated plates. The down‐regulation of Rictor significantly disrupted the formation of VM channels by A375 and MUM‐2B cells, as evidenced by most tubes remaining open and an increase in dissociative cells (Fig. 2B). The results generated from shRictor#1 are shown in Figure S1. This finding indicates that Rictor plays an important role in VM.

### Knockdown of Rictor inhibits melanoma cells proliferation and arrests cells in G2/M phase of the cell cycle

**Figure 3:**
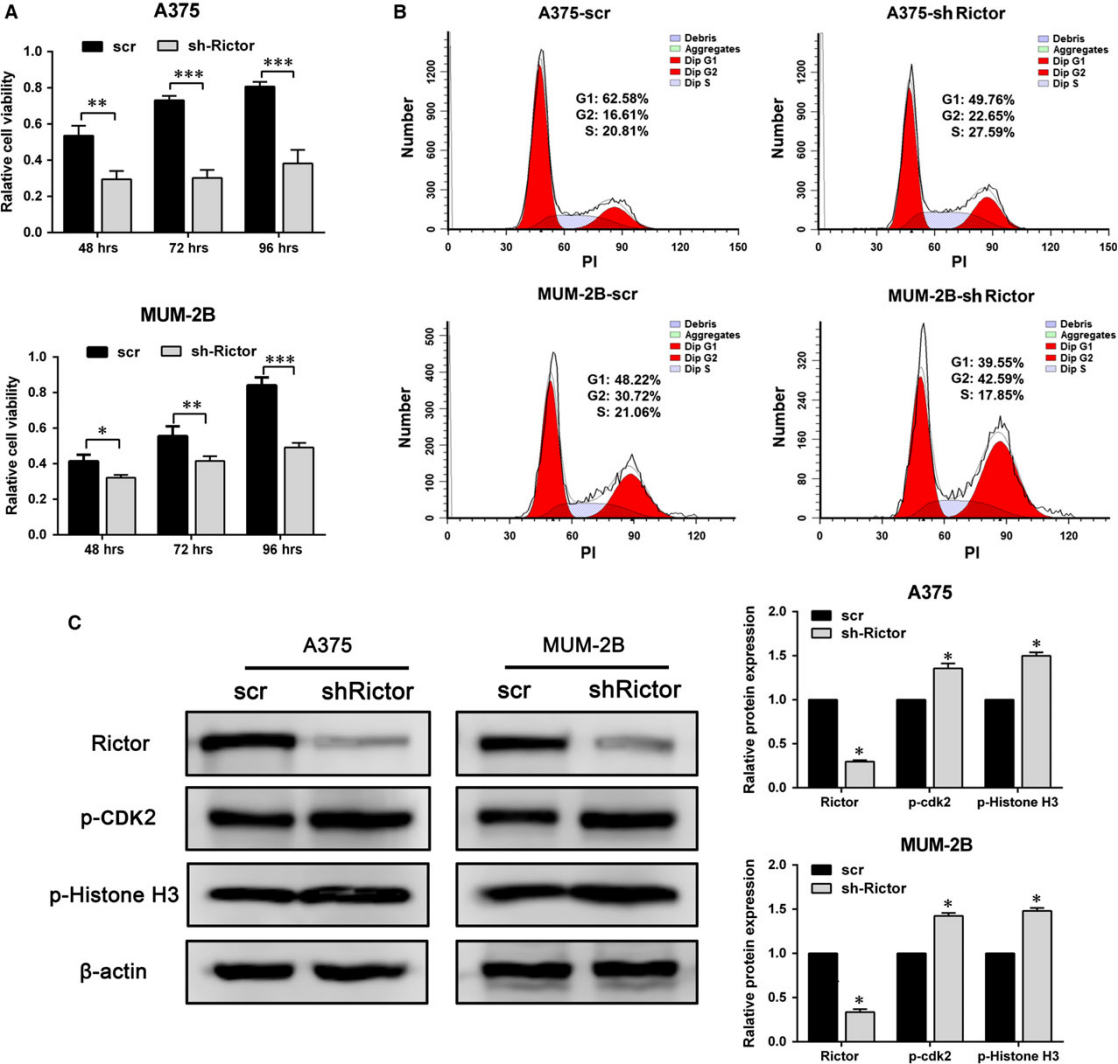
Knockdown of Rictor inhibits melanoma cells proliferation and blocked the cell cycle in G2/M phase. (**A**) Cell viability of A375 and MUM‐2B cells after Rictor knockdown evaluated by MTT assay (*P < 0.05, **P < 0.01). (**B**) Cell cycle of A375 and MUM‐2B cells after Rictor knockdown examined by FCM. (**C**) The expression of p‐CDK2 and p‐Histone H3 induced by knockdown of Rictor.

The proliferation and motility of melanoma cells are two essential elements for VM formation. Initially, A375 and MUM‐2B cell proliferation were evaluated using an MTT assay (Fig. 3A). This assay demonstrated that the down‐regulation of Rictor inhibited the proliferation of A375 and MUM‐2B cells, with decreases in viability of approximately 25% and 10% at 48 hrs, respectively, which did not interfere with the subsequent assays of motility properties. In addition, more than 40% and 60% of A375 and MUM‐2B cells, respectively, were alive even after prolonged exposure to 4 days, indicating that Rictor indeed affects, albeit slightly, the proliferation of melanoma cells. To further confirm the effect of Rictor on cell proliferation, FCM was applied to examine the cell cycle in A375 and MUM‐2B cells after Rictor knockdown. The results of this assay revealed that Rictor silencing enhanced the number of melanoma cells in G2/M phase and reduced the number of G0/S‐phase cells (Fig. 3B; shRictor#1 results as Fig. S2A). Besides, the expression of phospho‐CDK2 and phospho‐Histone H3.1 were assessed by Western blotting assays. The slightly increase of both cell cycle makers further confirmed the G2/M phase arrest induced by Rictor down‐regulation (Fig. 3C; shRictor#1 results as Fig. S2B).

### Knockdown of Rictor severely impairs melanoma cells migration, invasion and adhesion

**Figure 4:**
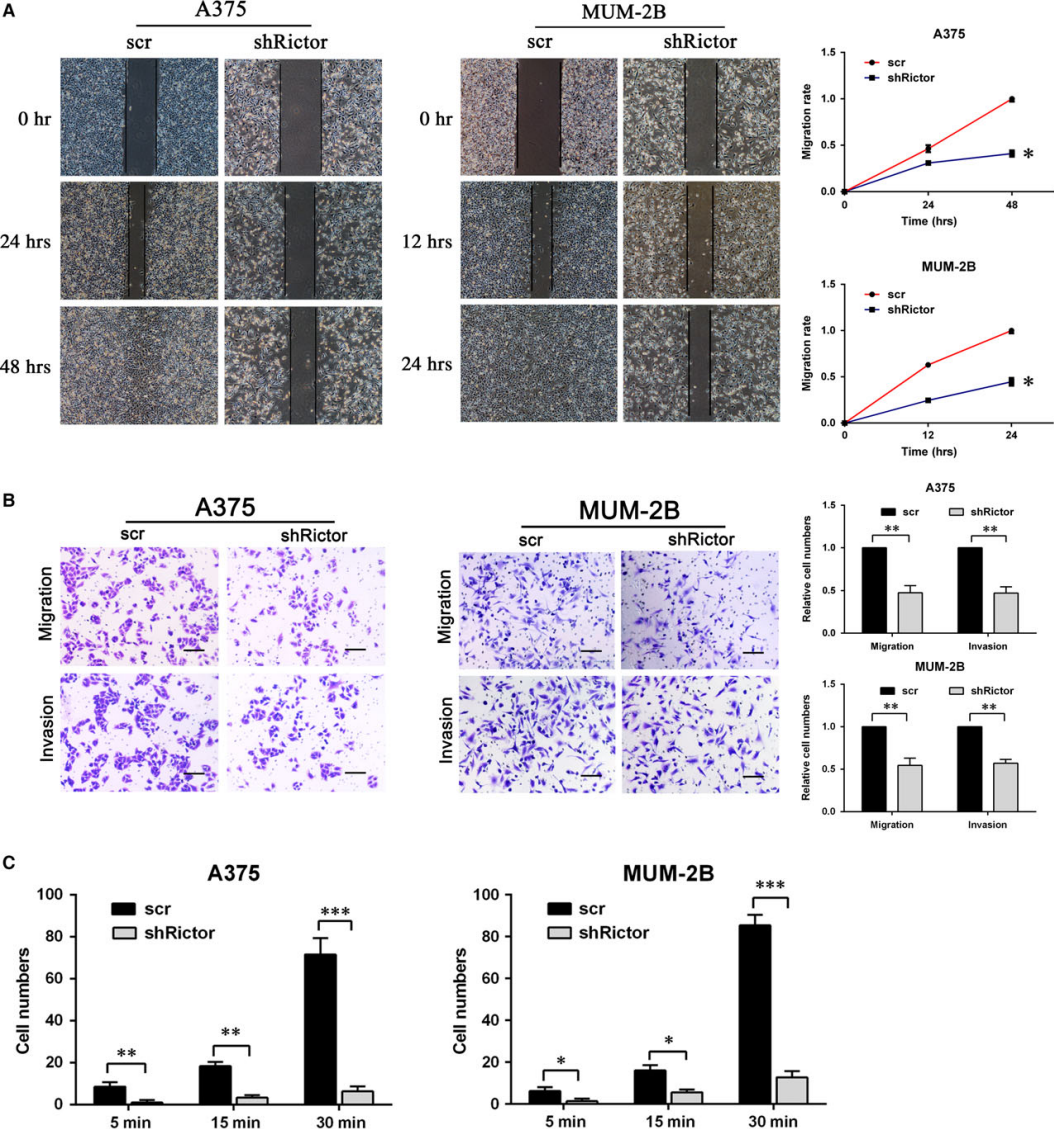
Knockdown of Rictor severely impaired A375 and MUM‐2B cells motility. (**A**) Migration of melanoma cells transfected with shRictor or scr in the wound healing assay (*P < 0.05). (**B**) Migration and invasion of A375 and MUM‐2B cells detected by transwell assay with or without Matrigel matrix (bar 100 μm, **P < 0.01). (**C**) adhesion assay of scr or shRictor melanoma cells (*P < 0.05, **P < 0.01, ***P < 0.001).

Migration, invasion and adhesion are required for the motility of tumour cells. The migration of melanoma cells was first evaluated with a wound‐healing assay. As shown in Fig. 4A, shRictor cells failed to heal the gap 24 hrs after the scratch, indicating that directional migration was inhibited. Cell motility was further evaluated with a transwell assay coated with Matrigel matrix to assess invasion or without to assess migration. After Rictor knockdown, the number of cells migrating through 8.0 μm PET decreased by approximately 55% (A375) and 45% (MUM‐2B) in the migration and invasion assays, respectively (Fig. 4B). In addition, Rictor down‐regulation significantly reduced the number of cells adhering to a coated coverslip at various time‐points during the adhesion assay (Fig. 4C). The results of shRictor#1 are as shown in Figure S3. Taken together, these data show that Rictor knockdown significantly impaired the motility of melanoma cells.

### Down‐regulation of Rictor impairs MMP‐2/9 expression and activity by inhibiting AKT activation

**Figure 5:**
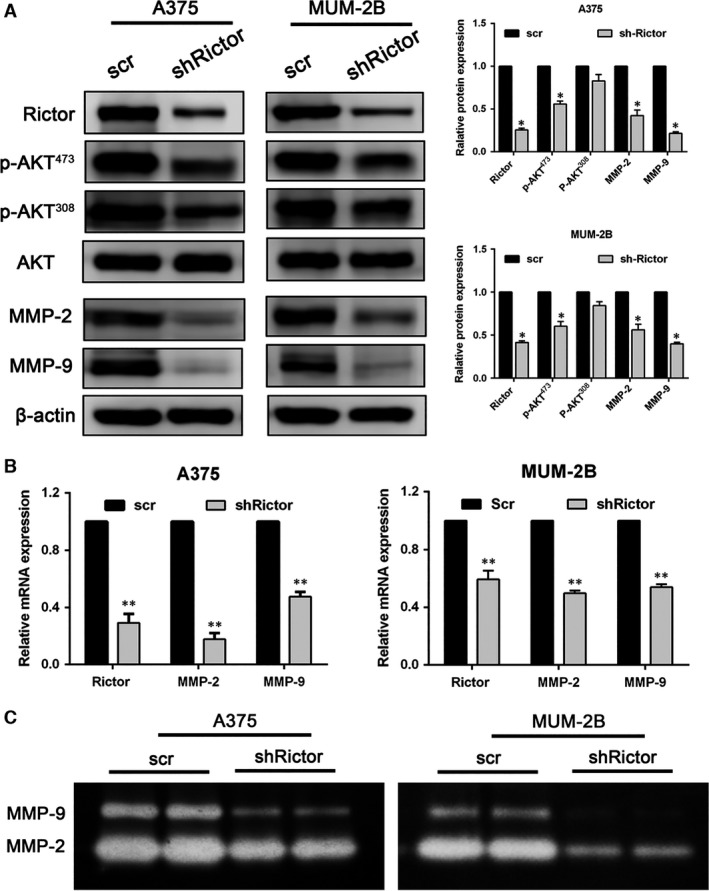
Down‐regulation of Rictor impaired MMP‐2/9 expression and activity by inhibiting the activation of AKT. (**A**) The expression of Rictor correlates with the phosphorylation of AKT Ser^473^ and Thr^308^ and the expression of MMP‐2/9 as detected by Western blotting assay (*P < 0.05). (**B**) RT‐PCR revealed that Rictor silencing induced down‐regulation of MMP‐2 and MMP‐9 at the mRNA level (**P < 0.01). (**C**) The activity of secreted MMP‐2 and MMP‐9 as assessed by Zymography assay.

The full activation of AKT depends on phosphorylation at Ser^473^ and Thr^308^, which are phosphorylated by Rictor‐mTORC2 and PDK1 19, respectively. Phosphorylated Ser^473^ serves as a docking site for PDK1 to phosphorylate Thr^308^ 34, 35. As shown in Figure 5A (Fig. S4A), Rictor knockdown severely impaired the phosphorylation of AKT Ser^473^, and the phosphorylation of AKT Thr^308^ was also affected. The expression of MMP‐2 and MMP‐9 was significantly inhibited due to AKT inactivation induced by the knockdown of Rictor, which was further confirmed at the mRNA level using real‐time PCR. Concurrent with the decrease in Rictor mRNA, the mRNA levels of MMP‐2 and MMP‐9 decreased approximately 80% (in A375 cells), 50% (in MUM‐2B cells) and 45% (in A375 cells), 30% (in MUM‐2B cells), respectively (Fig. 5B; shRictor#1 results as Fig. S4B). Furthermore, the activities of MMP‐2 and MMP‐9 were measured by gelatin Zymography. As shown in Figure 5C, the down‐regulation of Rictor severely impaired the activities of MMP‐2 and MMP‐9 secreted into the supernatant by both A375 and MUM‐2B cells.

### shRictor and the AKT inhibitor MK‐2206 exhibit similar inhibition of AKT‐MMP‐2/9

**Figure 6:**
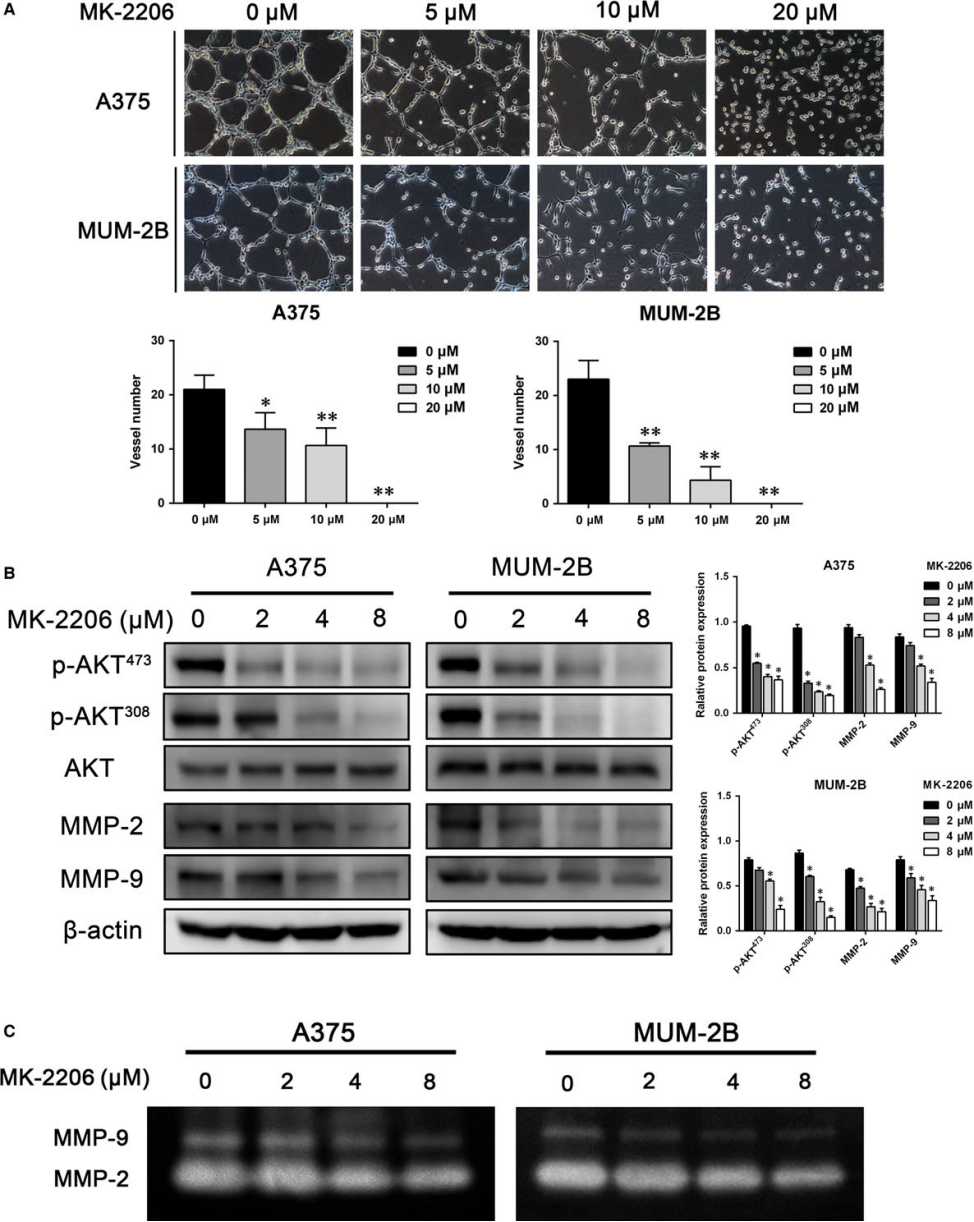
The inhibition of AKT‐MMP‐2/9 by shRictor was consistent with that by the AKT inhibitor MK‐2206. (**A**) treatment with MK‐2206 disrupted the channels formation in 3D cultures (**P < 0.05,**P < 0.01). (**B**) MK‐2206 induced a decrease in the phosphorylation of AKT Ser^473^ and Thr^308^ and the expression of MMP‐2/9 (*P < 0.05). (**C**) the activity of MMP‐2 and MMP‐9 secreted by cells treated with different concentrations of MK‐2206 was detected by Zymography assay.

To confirm that the effect of Rictor on MMP‐2/9 is mediated by AKT, a small‐molecule inhibitor of AKT, MK‐2206 32, was used to treat melanoma cells. As shown in Figure 6A, treatment with MK‐2206 significantly disrupted the channels formation by A375 or MUM‐2B cells and 20 μM of MK‐2206 completely inhibited the channels formation. The results suggest the direct role of AKT in VM. After treatment for 24 hrs, the phosphorylation of AKT at Ser^473^ was significantly inhibited, and the phosphorylation at Thr^308^ was also impaired; this inhibition was similar to that induced by Rictor knockdown. The expression levels of MMP‐2 and MMP‐9 severely decreased after AKT inhibition (Fig. 6B). Gelatin Zymography assay results revealed that the activities of secreted MMP‐2 and MMP‐9 were also significantly impaired by MK‐2206 (Fig. 6C). The effect induced by MK‐2206 was consistent with that induced by shRictor.

## Discussion

Melanoma remains one of the most lethal cancers, especially when accompanied by VM formation, and traditional anti‐angiogenesis therapy cannot inhibit VM lumen‐formation by aggressive cutaneous and uveal melanoma cells 9. Our results support the hypothesis that Rictor plays an important role in the VM formation of melanoma cells *via* the AKT‐MMP‐2/9 pathway. The pathological investigation showed that melanoma tissues overexpressing Rictor are prone to form VM channels, and this formation is accompanied by AKT membrane translocation and an increase in MMP‐2/9 secretion. Three‐dimensional cultures *in vitro* showed that down‐regulating Rictor with shRNA severely impaired the formation of VM tubes by A375 and MUM‐2B cells, further supporting our hypothesis that Rictor regulates VM formation.

Previous studies have suggested that the knockdown of Rictor inhibits the proliferation 36 and motility, including migration, invasion and adhesion, of breast cancer cells 30 and renal cell carcinoma 16, which are two key elements for the formation of VM channels by tumour cells. In this study, down‐regulating Rictor by shRNA affected the proliferation of A375 and MUM‐2B cells and arrested these cells in G2/M phase of the cell cycle, probably because the activation of AKT, which is involved in cell proliferation, survival and metabolism 22, was impaired, which is regulated by the Rictor/mTORC2 complex 20. Taken together, the results of the wound‐healing and Transwell assays showed that the migration and invasion of melanoma cells were significantly inhibited by Rictor knockdown, in accordance with Zhang's report 30 on breast cancer cells; both of these elements are required for VM formation. The function of Rictor is probably realized through AKT acting on its downstream effectors.

The Rictor/mTORC2 complex phosphorylates AKT at Ser^473^, which is a docking site for PDK1 to phosphorylate Thr^308^ 34, 35. In addition, MMP‐2 and MMP‐9 serve as important downstream effectors of AKT 10, 23, 24 and are involved in cell motility and VM formation 10, 26. Therefore, the hypothesis that Rictor regulates VM *via* the AKT‐MMP‐2/9 pathway is reasonable. As demonstrated above, Rictor silencing severely inhibited the phosphorylation of AKT at Ser^473^ and also affected the phosphorylation at Thr^308^, which inactivated AKT. As a result, the expression and activity of MMP‐2/9 significantly decreased, which may disrupt VM tubes *in vitro*. Moreover, the regulatory mechanism through which VM were modulated by Rictor in melanoma cells was consistent with AKT inhibition by MK‐2206, the first allosteric small‐molecule inhibitor of AKT to enter clinical development 32, 33. This result confirmed that AKT functioned as a bridge between Rictor and MMP‐2/9. This study is the first to demonstrate that Rictor plays an important role in melanoma VM formation by regulating the phosphorylation of AKT and its downstream effectors, MMP‐2/9, indicating that Rictor may be a potential biomarker for VM formation in melanoma and can be used to predict the prognosis of melanoma patients.

## Conflict of interest

The authors confirm that there are no conflicts of interest.

## Supporting information


**Figure S1** Rictor down‐regulation with shRictor#1 inhibited VM formation on Matrigel by A375 and MUM‐2B cells (**P<0.01).
**Figure S2** Knockdown of Rictor by shRictor#1 blocked cell cycle in G2/M phase.
**Figure S3** Knockdown of Rictor with shRictor#1 severely impaired A375 and MUM‐2B cells motility.
**Figure S4** Down‐regulation of Rictor with shRictor#1 impaired MMP‐2/9 expression and activity through inhibiting activation of AKT.
