# Supplementary material for: Rictor regulates the vasculogenic mimicry of melanoma via the AKT‐MMP‐2/9 pathway
Source: J Cell Mol Med. 2017 Jul 12;21(12):3579–91. doi: 10.1111/jcmm.13268 (PMC5706568; doi:10.1111/jcmm.13268)
Supplement: Supplementary file 1 — Figure S1 Rictor down‐regulation with shRictor#1 inhibited VM formation on Matrigel by A375 and MUM‐2B cells (**P<0.01). Figure S2 Knockdown of Rictor by shRictor#1 blocked cell cycle in G2/M phase. Figure S3 Knockdown of Rictor with shRictor#1 severely impaired A375 and MUM‐2B cells motility. Figure S4 Down‐regulation of Rictor with shRictor#1 impaired MMP‐2/9 expression and activity through inhibiting activation of AKT. [file JCMM-21-3579-s001.doc]

SUPPLEMENTARY INFORMATION：

**Rictor regulates the vasculogenic mimicry of melanoma via the AKT-MMP-2/9 pathway**

Xingmei Liang^#^, Ran Sun^#^, Xiulan Zhao, Yanhui Zhang, Qiang Gu, Xueyi Dong, Danfang Zhang, Junying Sun, Baocun Sun^*^


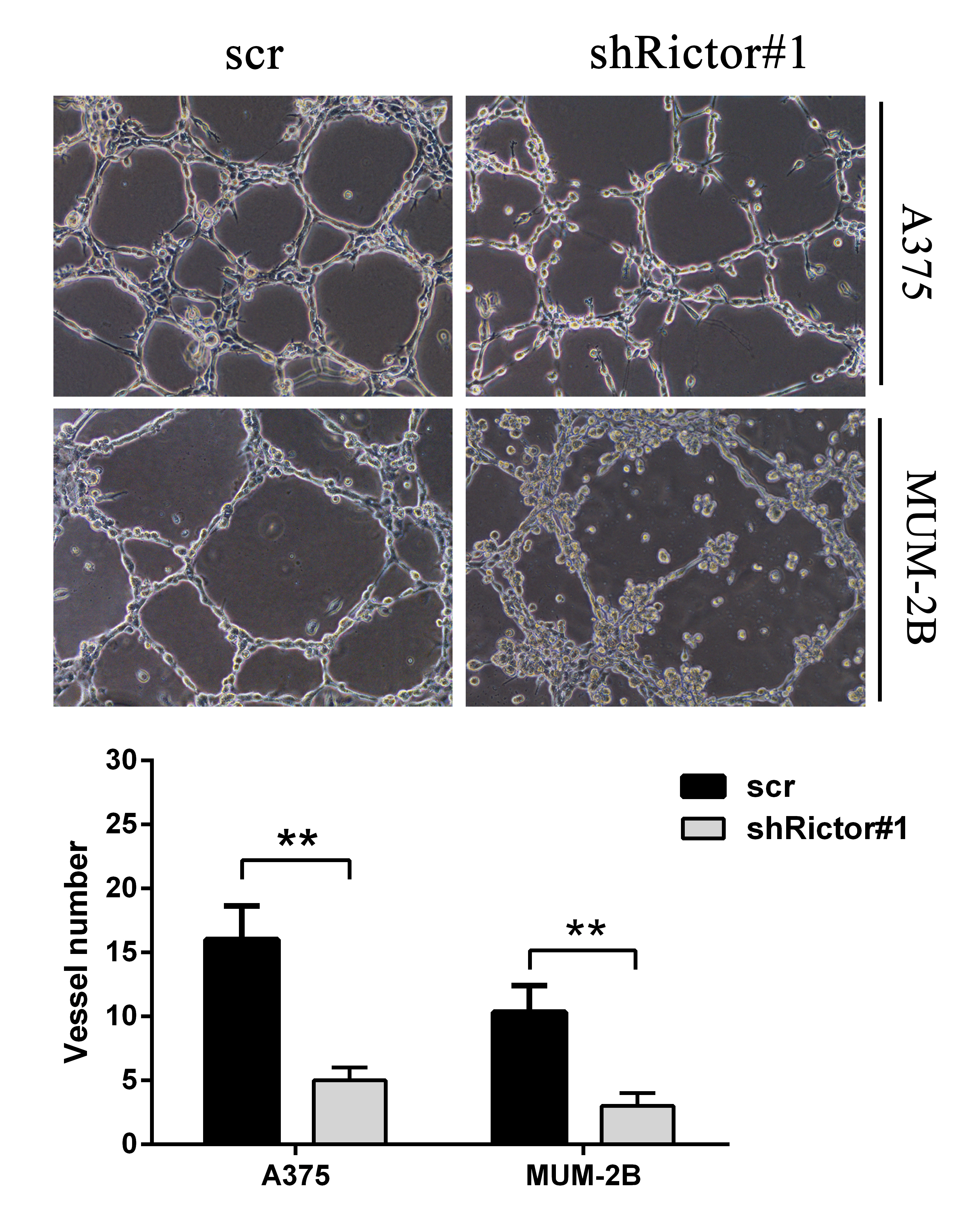


Fig. S1 Rictor down-regulation with shRictor#1 inhibited VM formation on Matrigel by A375 and MUM-2B cells (**p<0.01).


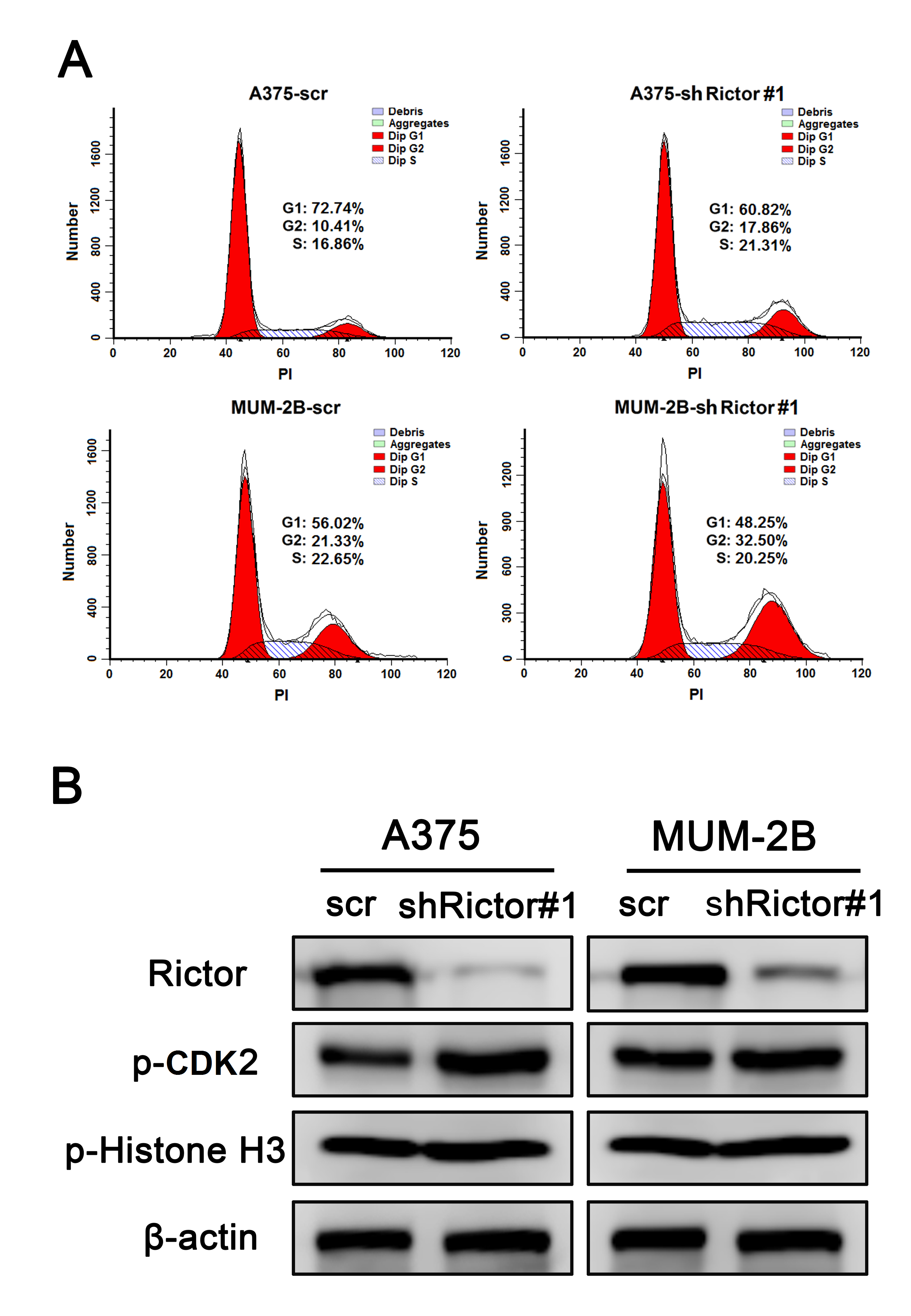


Fig. S2 Knockdown of Rictor by shRictor#1 blocked cell cycle in G2/M phase. A, Cell cycle of A375 and MUM-2B cells after Rictor knockdown examined by FCM. B, The expression of p-CDK2 and p-Histone H3 induced by transfection with shRictor#1.


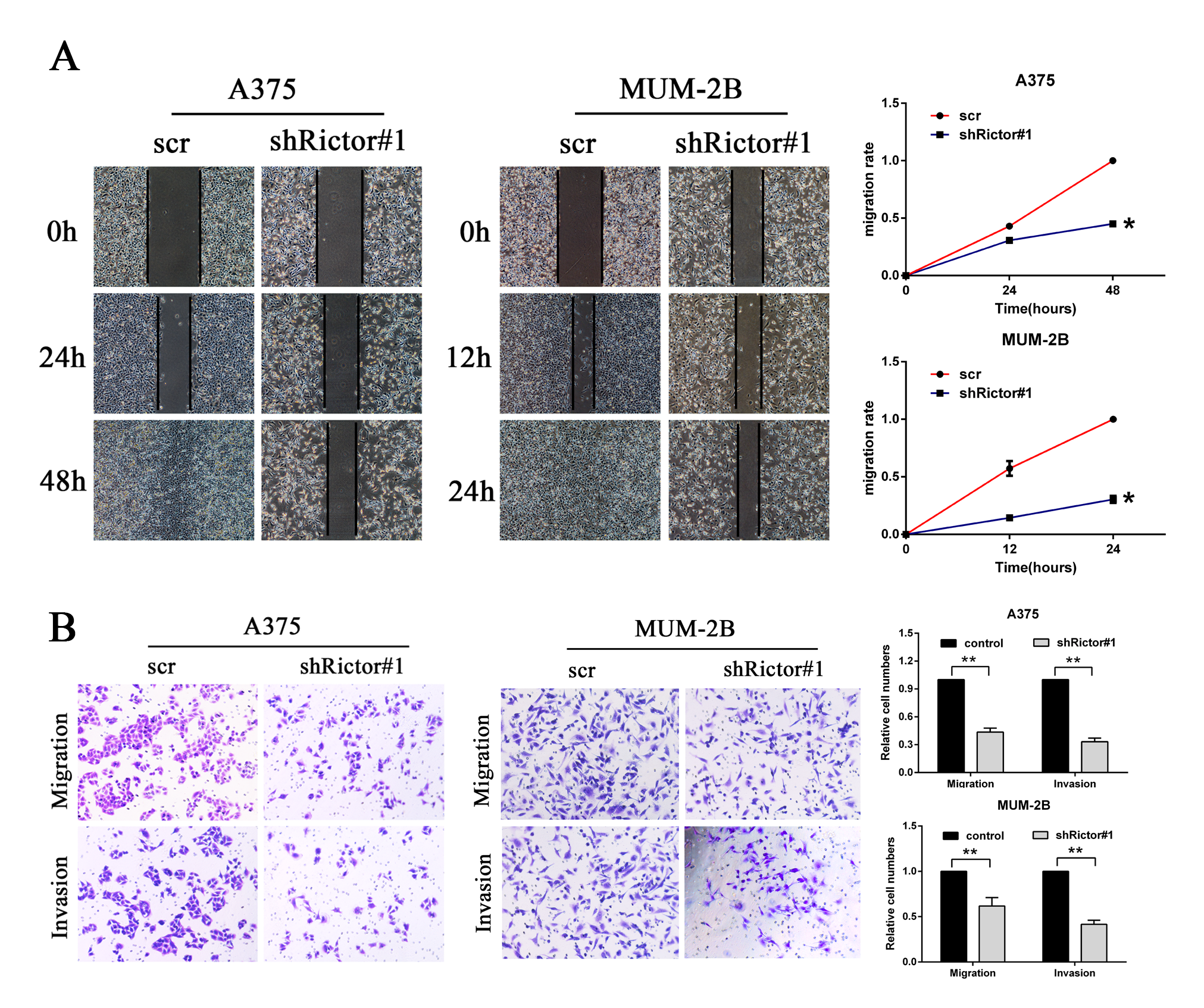


Fig. S3 Knockdown of Rictor with shRictor#1 severely impaired A375 and MUM-2B cells motility. A, Migration of melanoma cells transfected with shRictor#1 or scr in wound healing assay (*p<0.05). B, Migration and invasion of A375 and MUM-2B cells detected by Transwell assay with or without Matrigel matrix (**p<0.01).


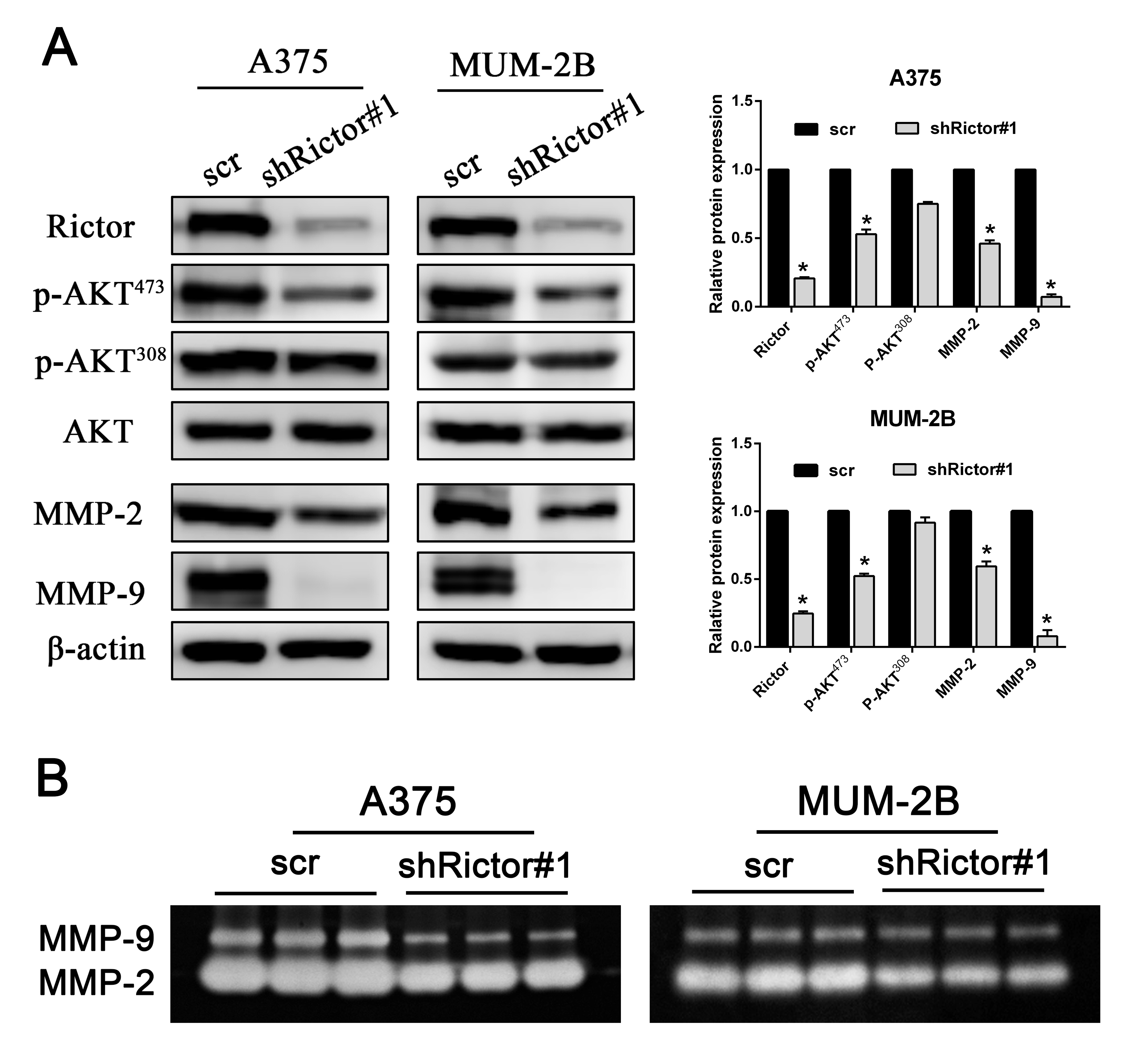


Fig. S4 Down-regulation of Rictor with shRictor#1 impaired MMP-2/9 expression and activity through inhibiting activation of AKT. A, The expression of Rictor correlates with the phosphorylation of AKT Ser^473^ and Thr^308^ and the expression of MMP-2/9 detected by western blotting assay (*p<0.05). B, The activity of secreted MMP-2 and MMP-9 tested by Zymography assay.
